# Wound Healing and Antioxidant Capabilities of *Zizyphus mauritiana* Fruits: In-Vitro, In-Vivo, and Molecular Modeling Study

**DOI:** 10.3390/plants11111392

**Published:** 2022-05-24

**Authors:** Nourhan Hisham Shady, Raya Soltane, Sherif A. Maher, Entesar Ali Saber, Mahmoud A. Elrehany, Yaser A. Mostafa, Ahmed M. Sayed, Usama Ramadan Abdelmohsen

**Affiliations:** 1 Department of Pharmacognosy, Faculty of Pharmacy, Deraya University, Universities Zone, New Minia City 61111, Egypt; norhan.shady@deraya.edu.eg; 2 Department of Basic Sciences, Adham University College, Umm Al-Qura University, Makkah 21955, Saudi Arabia; rasoltan@uqu.edu.sa; 3 Department of Biology, Faculty of Sciences, Tunis El Manar University, Tunis 1068, Tunisia; 4 Department of Biochemistry, Faculty of Pharmacy, Deraya University, Universities Zone, New Minia City 61111, Egypt; sherif.ali@deraya.edu.eg (S.A.M.); mahmoud.elrehany@deraya.edu.eg (M.A.E.); 5 Department of Histology and Cell Biology, Faculty of Medicine, Minia University, Minia 61519, Egypt, Delegated to Deraya University, Universities Zone, New Minia City 61111, Egypt; entesar.ali@deraya.edu.eg; 6 Department of Biochemistry, Faculty of Medicine, Minia University, Minia 61519, Egypt; 7 Pharmaceutical Organic Chemistry Department, Faculty of Pharmacy, Assiut University, Assiut 71526, Egypt; yaabdelkarem@pharm.aun.edu.eg; 8 Department of Pharmacognosy, Faculty of Pharmacy, Nahda University, Beni-Suef 62513, Egypt; ahmed.mohamed.sayed@nub.edu.eg; 9 Department of Pharmacognosy, Faculty of Pharmacy, Minia University, Minia 61519, Egypt

**Keywords:** *Zizyphus*, LC-MS profiling, wound healing, antioxidant, molecular docking

## Abstract

LC-HRMS-assisted chemical profiling of *Zizyphus mauritiana* fruit extract (ZFE) led to the dereplication of 28 metabolites. Furthermore, wound healing activity of ZFE in 24 adult male New Zealand Dutch strain albino rabbits was investigated in-vivo supported by histopathological investigation. Additionally, the molecular mechanism was studied through different in-vitro investigations as well as, studying both relative gene expression and relative protein expression patterns. Moreover, the antioxidant activity of ZFE extract was examined using two in-vitro assays including hydrogen peroxide and superoxide radical scavenging activities that showed promising antioxidant potential. Topical application of the extract on excision wounds showed a significant increase in the wound healing rate (*p* < 0.001) in comparison to the untreated and MEBO^®^-treated groups, enhancing *TGF-β1*, VEGF, Type I collagen expression, and suppressing inflammatory markers (*TNF-α* and *IL-1β*). Moreover, an in silico molecular docking against TNFα, TGFBR1, and IL-1β showed that some of the molecules identified in ZFE can bind to the three wound-healing related protein actives sites. Additionally, PASS computational calculation of antioxidant activity revealed potential activity of three phenolic compounds (Pa score > 0.5). Consequently, ZFE may be a potential alternative medication helping wound healing owing to its antioxidant and anti-inflammatory activities.

## 1. Introduction

Normally, successful wound healing process occurs in a proper sequence and time frame. Complicated clinical difficulties usually rise-up upon impairing this process at any of its four programmed phases (haemostasis, inflammation, proliferation, and remodelling) [1]. Many wound care products (MEBO^®^, Calmoseptine^®^, Boroline^®^, and others) and therapies have been developed and/or investigated as wound-healing process stimulants [1]. Broadly, traditional (especially herbal ones) and modern therapies are the two major classes that are widely used as wound healing. Interestingly, traditional herbal therapies still the preferable wound-healing therapies in rural populations of developing countries not only because of their availability and cheapness, but also because of their proven efficacy, clinical acceptance and low or no side effects as wound remedies. Herbal extracts of many plant species play a significant role in curing critical diseases [2,3,4,5,6,7,8,9] and have a great contribution to the wound healing process [10], such as the leaf extract of *Coccinia grandis* [11], curcumin obtained from the rhizomes of *Curcuma longa* [12], *Aloe vera*, aqueous extract of leaves of *Hippophae rhamnosides* and *Rosmarinus officinalis* L. Furthermore, both *Aloe vera* and *Calendula officinal* extracts have been reported to exhibit a good effect in the wound healing process [13,14]. All these herbal extracts commonly have at least one or more of active constituents (such as triterpenoids, flavonoids, phenols, polyphenols, vitamins, alkaloids, and/or sterols) which known to help in promoting wound-healing process. *Ziziphus mauritiana*, belongs to the family Rhamnaceae, is found in deserts and wild temperate regions indigenous to India, Algeria, Egypt, and southern Africa [15]. *Z. mauritiana* is known by other names such as Ber, Indian jujube, Jujube, Desert apple, Indian plum, Malay apple, and Chinese apple [16]. *Ziziphus mauritiana* is a rich source of various natural metabolites including triterpenoids [17], flavonoids, cardiac glycoside [18,19], alkaloids [20], leucoanthocyanidins [21], and sterols [22]. Moreover, *Ziziphus* ripened fruits are rich reservoirs of vitamins such as thiamine, riboflavin, niacin, and ascorbic acid [16]. Additionally, *Z. mauritiana* was used as a potent natural source for the treatment of different diseases along with its nutritional value. The fruits of *Z. mauritiana* exert a myriad of biological activities such as antioxidant [16], antidiabetic [23] activities. *Z. mauritiana* bark extract exhibited anti-inflammatory [24], anticancer and anti-allergic potential [25]. *Z. lotus* and *Z. jujuba* alcoholic extracts were reported to have interesting wound healing potential [26,27].

According to these proved and promising facts about wound-healing traditional herbal therapies and on the wound healing potential of *Ziziphus* fruits species, we decided to explore the wound healing potential and antioxidant capabilities of the *Z. mauritiana* fruits (which are widely-distributed in Egypt). These capabilities will be explored in this study through the following three avenues: (i) Investigating the antioxidant activity (in-vitro) and wound-healing activity (in-vivo) of *Z. mauritiana* fruits; (ii) Examining the correlation between the phytochemicals of the fruit’s extract, which were putatively characterized by the aid of LC-HRMS analysis, and their wound healing and antioxidant activities; and finally, (iii) carrying out virtual molecular docking experiments against relevant wound-healing proteins, in addition to theoretical prediction of antioxidant activity using predication of activity spectra of substances (PASS) online platform.

## 2. Materials and Methods

### 2.1. Plants Material

The fruits of *Z. mauritiana* were collected in April 2021 from Minia Governorate, Egypt. Samples were authenticated by Abdallah Salem, Minia, Egypt. A voucher specimen (ZM 1-2021) was archived at the Pharmacognosy Department, Faculty of Pharmacy, Deraya University, Egypt.

### 2.2. In-Vitro Antioxidant Activity

Hydrogen Peroxide Scavenging and Superoxide Radical Scavenging Activities of *Z. mauritiana* fruits crude extract were discussed in supplementary data in detail [3,28].

### 2.3. In-Vivo Wound Healing Activity

In this case, 24 adult male New Zealand Dutch strain albino rabbits (6 months with an average body weight ranging between 1 to 1.2 kg) were used. Rabbits were kept in separate cages at room temperature on standard diet with a 12 h dark and light cycle. The anesthetized rabbits were divided into 3 groups: Group 1 (untreated or bare wound), Group 2 (ZFE-treated), and Group 3 (MEBO^®^ “pure herbal, natural in origin, containing beta-sitosterol of *Phellodendron amurense*, *Scutellaria baicalensis*, *Coptis chinensis*, pheretima aspergillum, Beeswax and sesame oil” ointment-treated) and were depilated on the paravertebral area before wound creation by biopsy punch standard protocol in both epidermis and dermis layers (size and shape of wounds and other wound characteristics discussed in details in supplementary part), and wound was covered with standard surgical dressing and observed daily in the 3 groups for 14 consecutive days. The wound healing potency of *Z. mauritiana* fruits crude extract was assessed utilizing the excision wound model; tissue histopathology, gene expression, and western blotting in (see supplementary data).

### 2.4. LC-MS Analysis

LCMS was carried out using a Synapt G2 HDMS quadruple time-of-flight hybrid mass spectrometer (Waters, Milford, CT, USA), the details of the metabolomics analysis were discussed in the supplementary section [29,30,31].

### 2.5. Molecular Docking

In-Silico molecular docking of the identified compounds of ZFE against three different wound-healing related proteins was performed. 2AZ5 (PDB deposited crystal structure resolution was 2.10 Å) was used as a PDB entry for tumor necrosis factor-alpha (TNFα) since it was co-crystallized with a small molecule inhibitor capable of inhibiting TNFα activity in biochemical and cell-based assays. The second PDB used was 6B8Y (PDB deposited crystal structure resolution was 1.65 Å) as an entry for transforming growth factor-beta receptor (TGFBR1) co-crystallized with a heterobicyclic inhibitor, and finally 6Y8M (PDB crystal structure resolution was 1.90 Å) as an entry for interleukin 1 beta (IL-1ß) co-crystallized with its potent inhibitor, SX2 (a heterocyclic succinamic acid derivative). Structures of isolated compounds were prepared in ChemDraw Ultra (v. 8, 2013) and were transferred as smiles to Builder software embedded in MOE 2014 software and their energy was minimized. Proteins structures’ were also prepared according to MOE LigX protocol and their structures were protonated at a cutoff value of 15 Å. Validation of docking process of 3 co-crystallized ligands and all test compounds identified from ZFE family were carried out by triangular placement method and London δG for rescoring 1 for only 10 retained docking poses of each compound. The resultant docking poses for each compound were examined and arranged according to their free binding energy value (kcal/mol) and docking accuracy resolution (RMSD; Å). Finally, 2D interactions for each pose were inspected individually for binding interactions and arranged in tabular form.

### 2.6. Prediction of the Antioxidant activity

To predict the antioxidant activity of the dereplicated compounds, their structures were virtually screened using PASS platform (http://way2drug.com/passonline/index.php (accessed on 14 April 2022)) as smile codes. Thereafter, the generated activity predictions were investigated for the antioxidant and/or radical scavenging activities. This neural networking-based screening platform applies a large-scale pharmacophore-based virtual screening using a huge number of compounds with more than 3000 different biological activities. The generated results were provided as Pa scores (Probably Active scores), where structures with Pa scores > 0.5 indicates a high probability to show antioxidant activity in-vitro, while the reverse is true for compounds’ Pa scores < 0.5.

## 3. Results and Discussion

### 3.1. In Vitro Antioxidant Activity

#### 3.1.1. Hydrogen Peroxide Scavenging Activity

**Figure 1:**
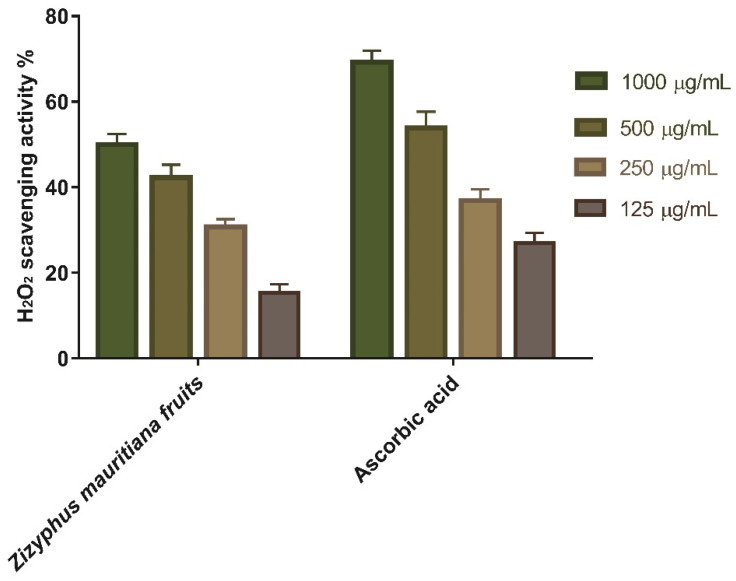
H_2_O_2_ Radical scavenging activity of *Z. mauritiana* fruit extract at different concentration (1000, 500, 250, and 125 µg/mL). Bars represent mean ± standard deviation (SD). The significant difference between test groups analyzed by a Two-way ANOVA test after normalization of variables by the Shapiro Wilk test.

Antioxidants are thought to manage wound oxidative stress and hence speed up the wound healing process. They play a critical role in controlling the damage of biological components such as DNA, protein, lipids, and body tissue that may sustain in the presence of reactive species. The antioxidant activity of ZFE, as a scavenger against H_2_O_2_, was investigated in this study. The maximal hydrogen peroxide radical scavenging activity of ZFE was 50 percent at 1000 µg/mL concentration, according to the data. ZFE suppressed the formation of hydrogen peroxide radicals in a dose-dependent manner, demonstrating a consistent antioxidant activity with IC_50_ of 189.2 μg/mL (Table 1 and Figure 1) and was compared with standard ascorbic acid (IC_50_ = 194.2 μg/mL). High levels of reactive oxygen species (ROS) in the wound site can promote collagen breakdown and hence the destruction of the extracellular matrix (ECM), which lead to marked reduction in processes such as angiogenesis and re-epithelialization, which are crucial for wounds to heal [32,33]. Moreover, elevated ROS can induce inflammation, increase pro-inflammatory cytokines, and hence prolong inflammation [34]. The antioxidant activity of ZFE that is attributed to its SOD activity and H_2_O_2_ scavenging activity, which can eliminate ROS and hence enhancing wound-healing process. These antioxidant properties found with ZFE extracts could be attributed to its phenolic content.

#### 3.1.2. Superoxide Radical Scavenging Activity 

**Figure 2:**
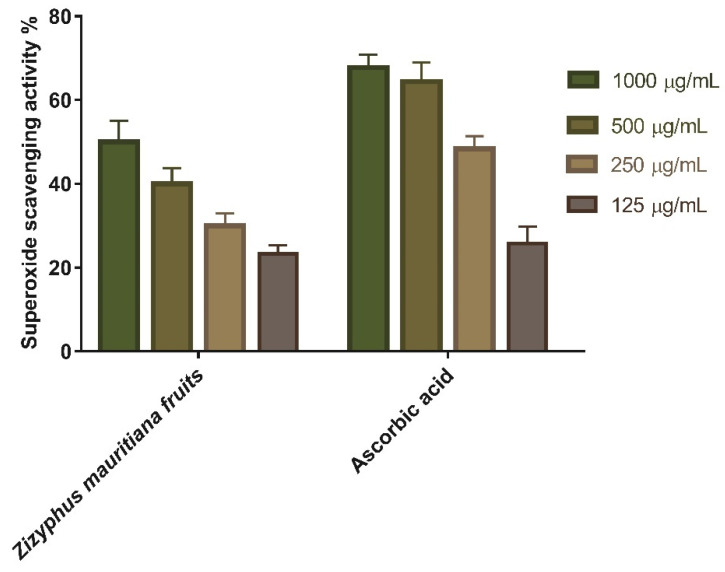
Superoxide radical scavenging activity of *Z. mauritiana* fruit extract at different concentration (1000, 500, 250, and 125 µg/mL). Bars represent mean ± SD (standard deviation). The significant difference between test groups analyzed by a Two-way ANOVA test after normalization of variables by the Shapiro Wilk test.

Redox signaling and enhanced oxidative stress play an important role in normal wound healing by encouraging hemostasis, inflammation, angiogenesis, granulation tissue creation, wound closure, and extracellular matrix development and maturation [35]. As a result, the superoxide scavenging activity of ZFE was evaluated, and the results revealed the scavenging impact of both ascorbic acid and ZFE extract. As shown in Table 2 and Figure 2, superoxide-scavenging activity rises almost linearly with concentration. Moreover, ZFE extract showed 50% superoxide scavenging efficacy at concentration of 1000 μg/mL. Finally, it worth mentioning that the concentration of ZFE needed for 50% inhibition (IC_50_) was found to be 146.7 µg/mL (c.f. 154.4 μg/mL for ascorbic acid).

### 3.2. Wound Healing Activity

#### 3.2.1. Wound Closure Rate Estimation

Wound healing is a complex process of repairing tissue structure in injured tissue and it contains three phases: an inflammatory process owing to pro-inflammatory-mediators secretion and immune system suppression, a proliferative phase via the proliferation of fibroblasts, collagen growth, and fresh blood vessels development as well as a remodeling phase that covers regeneration and injured tissue repair [35,36,37]. Wound closure can be represented as the centripetal flow of the edges of a full-thickness wound to aid the closure of the wound tissue [38,39,40]. Wound closure is thus an indicator of re-epithelialization, granulation, angiogenesis, fibroblast proliferation, keratinocyte differentiation, and proliferation [40]. MEBO^®^ as an internationally and widely used wound and burns-healing ointment have been proved to have anti-inflammatory and anti-microbial effect due to the presence of β sitosterol and berberine, respectively. Many studies have reported that MEBO provides suitable moist environment needed for burn wounds for optimal healing and re-epithelialization without the need for wound closure by dressing. In addition, some studies have proved the efficacy of MEBO in secondary healing of partial thickness wounds, such as split thickness skin graft sites, with improved cosmetic results and better scar quality.

**Figure 3:**
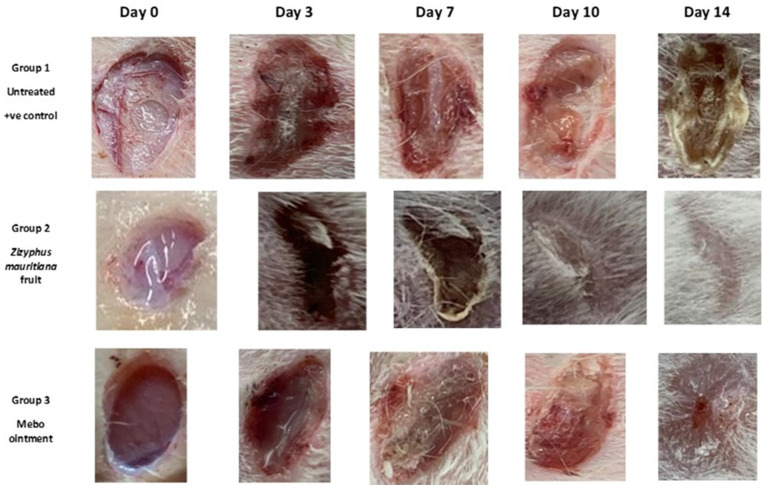
The excisional wounds on days 0, 3, 7, 10, and 14 post-wounding results of the activity for *Z. mauritiana* fruit extract and MEBO^®^ in wound of adult male New Zealand Dutch strain albino rabbits: Group 1: untreated (Negative control), Group 2: ZFE-treated group, and Group 3: MEBO^®^-treated group (Positive control).

Therefore, drugs that could accelerate wound repair with a potential input in all the process phases are preferred for efficient therapy, especially cheap ones with fewer side effects. The results showed a time-dependent increase in wound closure flow within all experimental groups. On the 3rd day post-injury, the wound closure rate was around 7 to 16% in each group, being the smallest in the untreated group and the highest in the treated ones, with no significant difference (*p* > 0.001) between groups. On the 7th day after treatment, the wound closure in the ZFE-treated group reached a 45%, which appeared to be significantly higher (*p* < 0.001) than the corresponding untreated group as shown in Figure 3.

**Figure 4:**
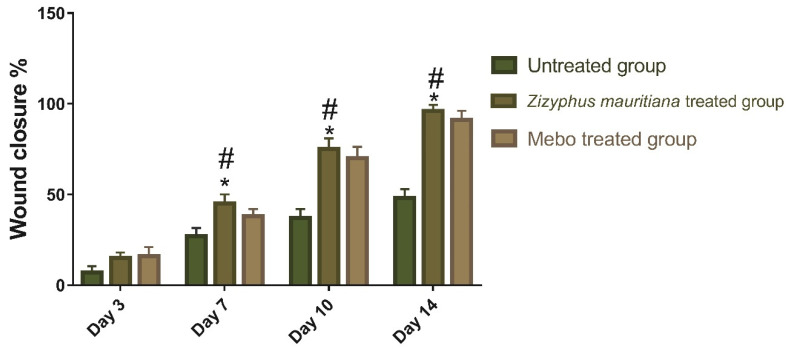
Wound closure rates: Group 1: untreated group (negative control), Group 2: ZFE extract-treated group, Group 3: MEBO^®^-treated group (positive control) over time post-injury (0, 3, 7, 10, and 14 days). The significant difference between groups analyzed by a Two-way ANOVA test after normalization of variables by the Shapiro Wilk test. Data were expressed as mean ± SD. * *p* < 0.001 compared with those of the untreated group on the respective day and *^#^ p* < 0.001 compared with those of the MEBO^®^ group on the respective day.

In addition, the ZFE-treated group also showed faster wound closure rates compared to the MEBO^®^-treated group (38%) (*p* < 0.001). Moreover, the wound closure rates on the 10th day post-burn were still significantly higher (*p* < 0.001) for the ZFE-treated group (70%) compared to the untreated group (37% wound closure rate). Finally, on the 14th day post-burn, the wounds in the ZFE-treated group were completely healed with a wound closure rate touching a 96% (cf. only 91% for the MEBO^®^-treated group), as shown in Figure 4.

#### 3.2.2. Effect of *Z. mauritiana* Fruit Extract on Expression of TGF-β, TNF-α, IL-1β, Collagen Type I, and VEGF

**Figure 5:**
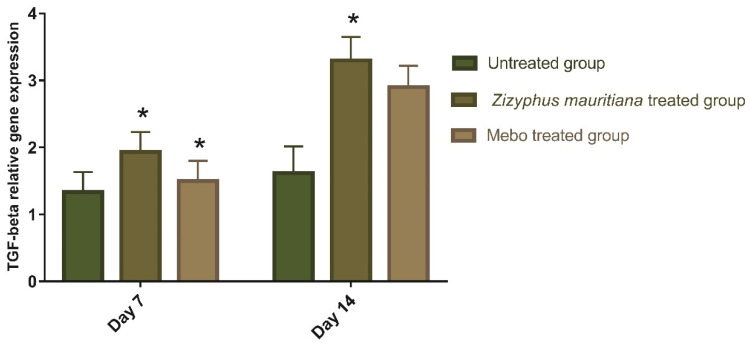
Gene expression in wound tissues for rabbits of different groups via quantitative RT-PCR. Data represent fold change relative to the normal control group expression after normalization to glyceraldehyde 3-phosphate dehydrogenase (GAPDH). Bars represent mean ± SD. The significant difference between groups analyzed by a two-way ANOVA test, where: * *p* < 0.001 compared to the untreated group on the respective day.

Wound-healing processes require complex interactions between cells as well as numerous growth factors [41], where the *TGF-β* hits the most crucial part throughout all phases of wound healing. During the hemostasis and inflammation phase, the *TGF-β* recruits and activates inflammatory cells, covering neutrophils and macrophages, whereas, in the proliferative phase, it creates multiple cellular responses having re-epithelialization, angiogenesis, granulation tissue development, and extracellular matrix deposition [41]. It stimulates fibroblasts to multiply and differentiate into myofibroblasts that participate in wound closure in the remodeling phase [42,43,44]. Chronic, non-healed wounds generally produce a failure of *TGF-β1* warning, while Feinberg and his co-worker declared that *TGF-β1* delivers an inhibitory effect on the expression of collagenases, which impair collagen and ECM [45]. As shown in Figure 5, the mRNA expression of *TGF-β* following excisional wound therapy with ZFE extract and MEBO^®^. *TGF-β* relative mRNA expression in skin tissues was substantially higher in ZFE-treated wounds on day 7 or even on day 14 compared to the untreated group (*p* < 0.001). The relative marker’s expression of ZFE-treated wounds was significantly higher than in the MEBO^®^-treated group. These notes are coherent with the above measurements, which established the ZFE enhanced *TGF-β1* expression and wound healing. Additionally, the mRNA expression in the wound tissue showed a remarkable rise in *TGF-β1* levels with ZFE-treated wound tissues compared to the untreated wound tissues (negative control).

**Figure 6:**
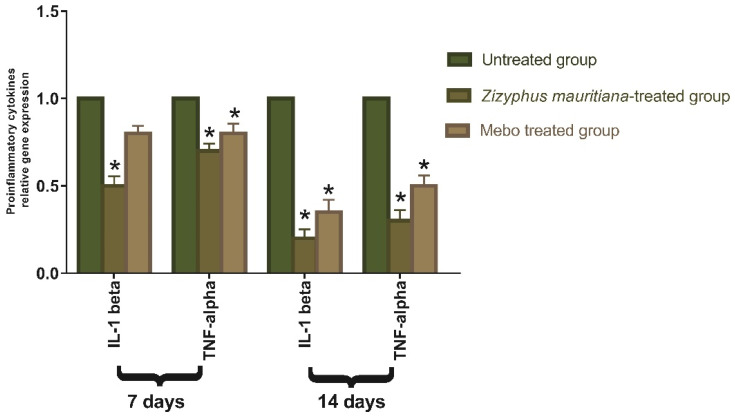
Quantitative RT-PCR analysis of genes expression in wound tissues of rabbits. Data represent fold change relative to the normal control group expression after normalization to glyceraldehyde 3-phosphate dehydrogenase (GAPDH). Bars represent mean ± SD. The significant difference between groups analyzed by a two-way ANOVA test, where: * *p* < 0.001 compared with those of the untreated group on the respective day.

*TNF-α* is one of the growth factors secreted from macrophages, which mixes with *IL-1β* to increase and suppress respective collagen production and fibroblast proliferation [46]. The *TNF-α* stimulates *NF-κB*, which in turn promotes gene expression of a plethora of pro-inflammatory cytokines including *TNF-α* itself and proteases, such as MMP, to free soluble *TNF-α* and potentiate the effects of this inflammatory cytokine [47]. Analysis of mRNA expression of full-thickness wound samples on day 7 post-injury revealed that the activity of the inflammatory markers *TNF-α* and *IL-1β* was significantly down-regulated in wounds treated with ZFE extract or MEBO^®^ compared to the untreated wounds, as shown in Figure 6. However, wounded rabbits treated with ZFE displayed a significantly much more reduction in the inflammatory markers (*TNF-α*, and *IL-1β*) compared to the MEBO^®^-treated group. Moreover, tissues treated with either ZFE or MEBO^®^ on day 14 still showing a significant decrease in both *TNF-α* and *IL-1β* mRNA expression compared to the untreated group at (*p* < 0.001). It is noteworthy to mention that, the expression of *TNF-α* and *IL-1β* in ZFE-treated wounds was markedly lower than in the MEBO^®^-treated group. Suitable expression of pro-inflammatory cytokines (*IL-1β* and *TNF-α*) is necessary to recruit neutrophils and exclude bacteria, and other contaminants from the wound site and this is recognized by dynamic inducers of Metalloproteinase (MMP) synthesis in inflammatory and fibroblasts cells. In wound healing, the MMP degrades and removes damaged ECM to aid wound repair [48]. However, a lengthy duration of the inflammatory phase leads to a problem in the healing process and these cytokines and proteinase damage the tissue and lead to the development of chronic wounds. So, suppressing inflammatory cytokines (*TNF-α*, and *IL-1β*) by ZFE can inhibit the ongoing inflammation and prevent wound repair impairment. These results suggested that ZFE could accelerate the switching process from an inflammatory to a non-inflammatory response with afterward increase in curing rate.

**Figure 7:**
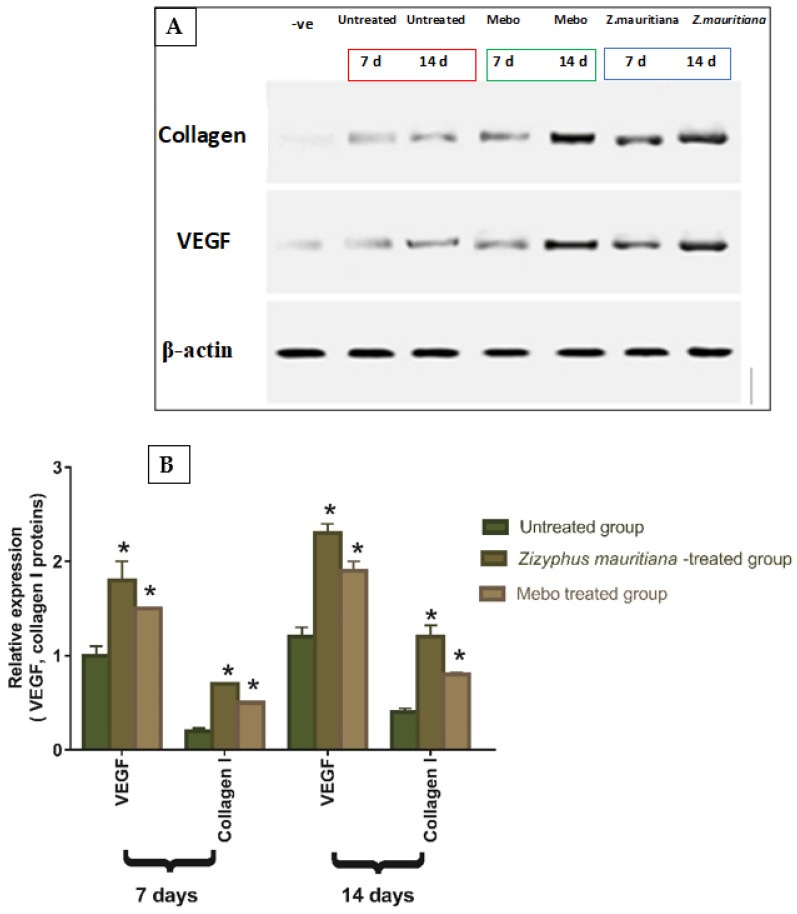
Effect of *Z. mauritiana* fruit extract on the expression of VEGF and collagen type I proteins: (**A**) Representative Immunoblotting of VEGF, collagen type I proteins, and β-actin proteins for all groups. (**B**) Expression of VEGF and collagen type I proteins, respectively, were expressed densitometrically (using bands) in after normalization to the corresponding internal control β-actin, as fold change relative to that of normal control rats. Bars represent mean ± SD. The significant difference between groups analyzed by a two-way ANOVA test, where: * *p* < 0.001 compared with those of the untreated group on the respective day.

Vascular endothelial growth factor (VEGF) hits a significant role in formation of new blood vessels [49], also, it stimulates wound healing via collagen deposition, angiogenesis, and epithelialization [50]. Moreover, wound repair is mediated by type I collagen, which is the main protein in skin tissue and shows an essential role in connective tissue repair by maintaining tissue health and an ECM structure for cellular adhesion and movement [51]. The task of collagen in wound healing is to attract fibroblasts and simplify the deposition of new collagen to the wound bed [52]. As shown in Figure 7, the relative protein expression of VEGF and type I collagen was illustrated. Analysis of the relative expression of VEGF and type I collagen in full-thickness wound samples on day 7 post-injury showed significantly up-regulated levels in wounds treated with ZFE or MEBO^®^ compared to the untreated wounds. However, wounded rabbits treated with ZFE displayed a significantly much more elevation in the relative protein expression compared to MEBO^®^-treated rabbits. Moreover, ZFE treatment or MEBO^®^ treatment for 14 days showed significant increase in relative protein expression when compared to untreated wounds at (*p* < 0.001). In addition, the relative expression of VEGF and type I collagen in ZFE-treated wounds was markedly higher than in MEBO^®^-treated wounds. These data are coherent with the early findings that ZFE enhanced VEGF expression and improved wound healing. The relative protein expression of VEGF was developed in ZFE-treated wound tissues related to untreated wound tissues, which might suggest that ZFE increased VEGF expression in wound tissues. 

#### 3.2.3. Histopathological Study

**Figure 8:**
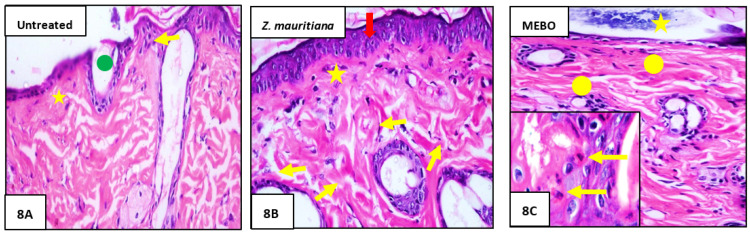
Histograms of wounded skin on day 7 after incision: (**A**) of Group 1 (untreated) showing the normal edge of the wound with the normal epidermis (yellow arrow) the wound is filled with blood clots (green circle) and underlying sloughed granulation tissue with compact and irregular collagen bundles (yellow star); (**B**) of Group 2 (*Z. mauritiana* fruits extract) showing marked re-epithelization (red arrow), granulation tissue filling the base of the defect from below is mainly cellular (star), and collagen bundles as appear as disorganized coarse and wavy bundles (yellow arrows). (**C**) of Group 3 (MEBO^®^-treated) showing scar tissue blocking the wound (star), Collagen bundles packing the defect in a reticular pattern resembling that of the adjacent normal dermis (circle). The inset shows inflammatory cellular infiltration mainly macrophages (yellow arrows). (Hematoxylin and eosin stain × 200 and 400).

Seven days after treatment, Group 1 (untreated group) showed normal wound’s edge with normal architecture features: having epidermis, well-formed dermal collagen bundles, hair follicles, and sebaceous glands. Additionally, the wound found filled with blood clots, sloughed granulation tissue with collagen fibers compactly organized in an irregular pattern, extravasated RBCs, and inflammatory cellular infiltration, and finally, the striated muscle showed necrotic myofiber in the deepest part of the wound (Figure 8A). 

In Group 2 (ZFE-treated group), the blood clot observed over the wound was still apparent, marked re-epithelization and the granulation tissue filling the defect from below was mainly cellular. In addition to, disorganized dense collagen with fibers appeared compactly arranged in an abnormal pattern resulting in distinct scarring in comparison to other treated groups (Figure 8B). Finally, Group **3** (MEBO^®^-treated group), scar tissue closing the wound and creeping of epidermal cells at wound edges were marked with a partial re-epithelization, and a marked inflammatory cellular infiltration (mainly of macrophages) and collagen fibers came packing the defect in a reticular pattern with a distance in between approximately resembling that of the neighbor’s natural dermis. Finally, the reticular dermis contained usual active, lengthened, and spindle-shaped fibroblasts with basophilic cytoplasm and open face oval nuclei (Figure 8C).

**Figure 9:**
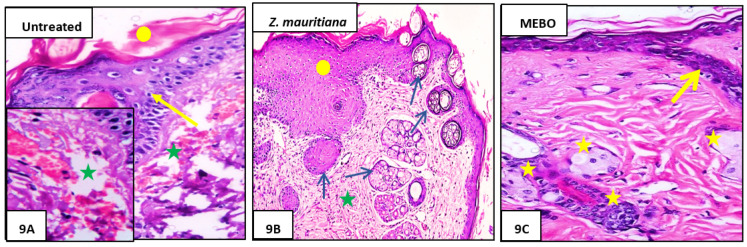
Histograms of wounded skin 14 days after incision: (**A**) of Group 1 (untreated) showing the wide wound area (yello circle), heavy inflammatory cellular infiltration in an acidophilic matrix (green star), and the normal skin (yellow arrow). (**B**) of Group 2 (*Z. mauritiana* fruits extract) showing the typical stratified squamous keratinized epithelium (yellow circle), dermal matrix with coarse wavy collagen bundles in different directions (green star), and numerous newly formed hair follicles (blue arrows). (**C**) of Group 3 (MEBO^®^-treated) showing typical epithelium with thin scar tissue extending into the dermis (yellow arrow), reticular dermis has coarse wavy collagen bundles arranged in different directions, and newly formed hair follicles (yellow stars). (Hematoxylin and eosin stain × 200 and 400).

In this case, 14 days after treatment, Group 1 (untreated group) developed a larger wound area and was packed with a heavy coat of granulation material, which was composed of several layers of connective tissue cells in an acidophilic matrix and overlying fat inflammatory cellular infiltration. The dermis is composed of confused, weak collagen with marked neovascularization (Figure 9A). 

Group 2 (*Z. mauritiana* fruits extract-treated group) showed contracted scar tissue blocking the wound and the epidermis appeared to form of only 1–3 rows of epithelial cells. The granulation tissue from below was mainly cellular and populated with fibroblasts, while the reticular layer contained disorganized dense compactly arranged collagen fibers (Figure 9B). 

Group 3 (MEBO^®^-treated groups), the skin tissue presented more or less normal with normal stratified squamous keratinized epithelium. Weak scar tissue may spread into the dermis. The dermal matrix showed many hair follicles, blood capillaries, and deficiency of inflammatory cellular infiltration. The collagen bundles in the papillary dermis are presented as fine interlacing bundles, and the reticular dermis is presented as coarse wavy bundles formed in various paths (Figure 9C).

### 3.3. LC-HRMS Chemical Profiling

**Figure 10:**
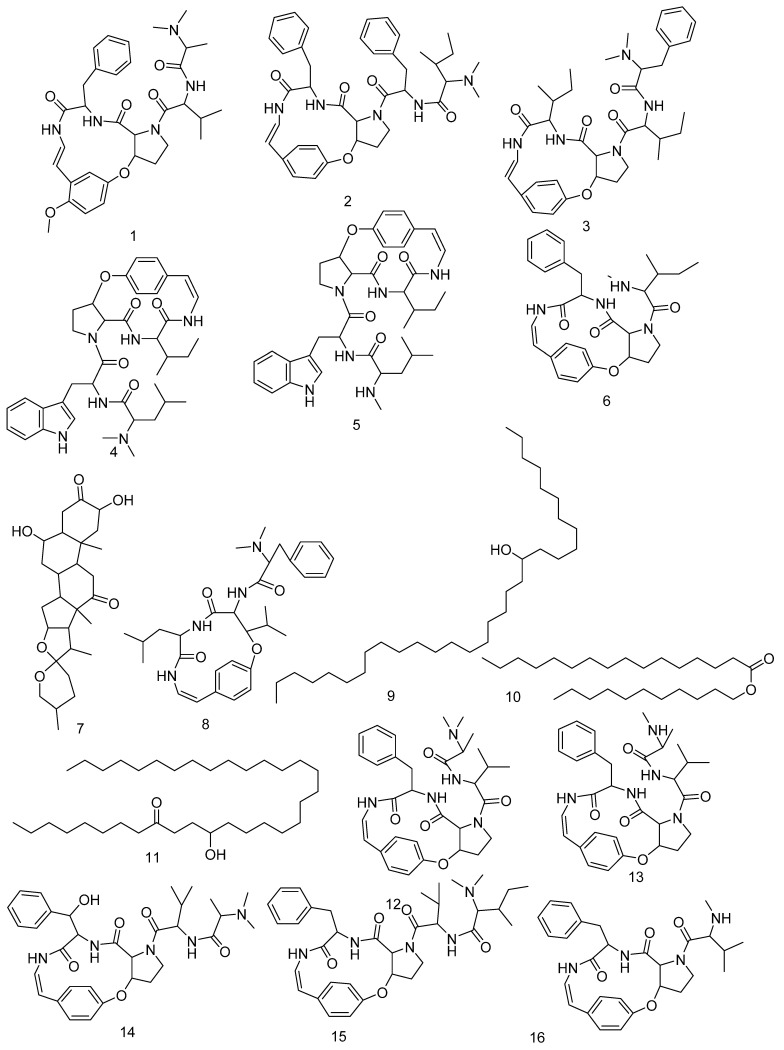

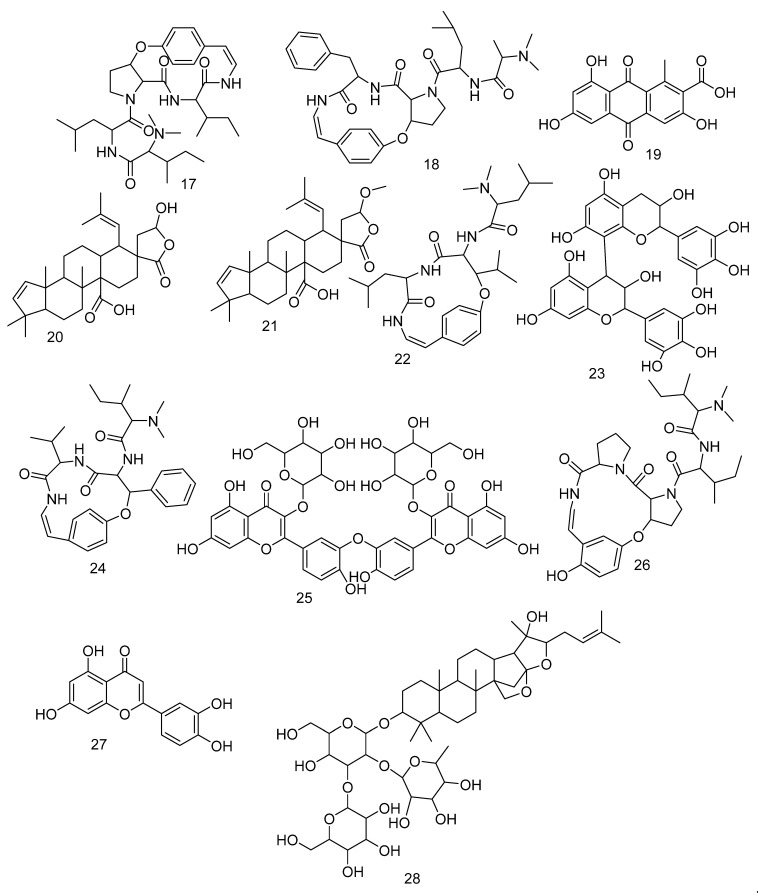
Compounds identified and dereplicated from ZFE extract.

LC-MS profiling for the crude ZFE was carried out to identify their chemical components, and identification of the annotated compounds was carried out depends on HR-ESIMS compared to literature data. Dereplicated compounds as shown in (Table S2 and Figure 10) belong to different phytochemical classes such as: amphibine H (**1**) [53], amphibine B (**2**) [54], amphibine D (**3**) [54], amphibine E (**4**) [54], mauritine J (**5**) [55], amphibine F (**6**) [56], zizogenin (**7**) [57], frangufoline (**8**) [56], 12-Hentriacontanol (**9**) [58], hexadecanoic acid undecyl ester (**10**) [59], 12-hydroxy-9-tetratriacontanone (**11**) [58], mauritine A (**12**) [57], mauritine F (**13**) [60], mauritine E (**14**) [61], mauritine B (**15**) [56], mauritine C (**16**) [56], mauritine D (**17**) [56], mauritine H (**18**) [60], 3,6,8-Trihydroxy-1-methylanthraquinone-2-carboxylic acid **(19)** [10], zizimauritic acid C (**20**) [62], zizimauritic acid C 21-Me ether (**21**) [62], franganine (**22**) [63], gallocatechin-(4α→8)-gallocatechin or prodelphinidin B (**23**) [64], sativanine A (**24**) [65], zizyflavoside B (**25**) [65], Zizyphine F (**26**) [53], 3′,4′,5,7-Tetrahydroxyflavone (**27**) [66], jujubasaponin IV (**28**).

### 3.4. Computational Analysis

#### 3.4.1. Molecular Docking Studies 

To further explore the wound healing potential of the identified compounds in ZFE extract, in-silico molecular docking experiments were performed using MOE^®^ program. The X-ray crystal structure of tumor necrosis factor with a small molecule inhibitor (TNF-alpha PDB ID: 2AZ5, resolution 2.10 Å) was used to construct a protein-ligand complex of various molecules isolated from ZFE, and obtained data were compared to the co-crystallized ligand as shown in Table 3. The binding free energy (in kcal/mol), binding interactions with various active site amino acid residues, and binding accuracy (RMSD) were used to predict binding modes and affinities of isolated molecules of ZFE.

**Figure 11:**
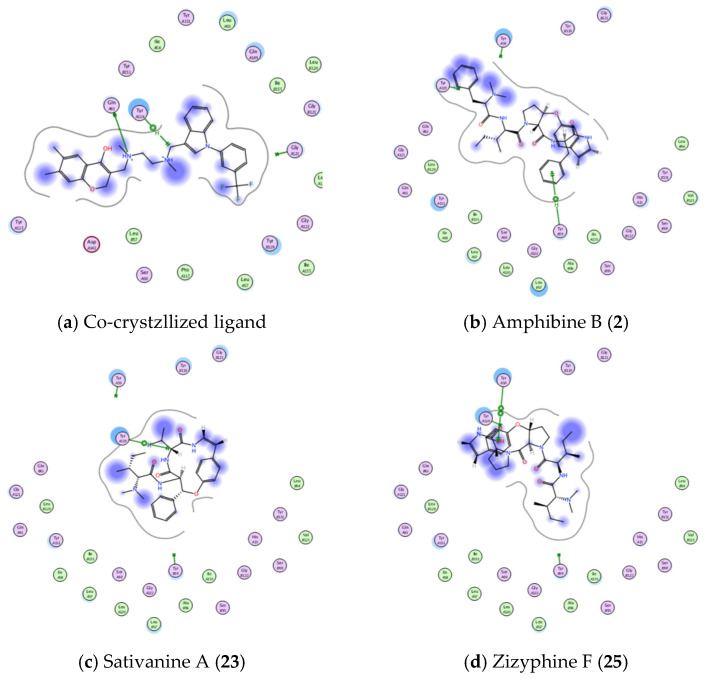
Showing 2D-binding interactions of co-crystallized ligand (**a**) and molecules **2**, **23**, **25** (**b**–**d**, respectively) within active sites of TNFα active site (PDB ID: 2AZ5).

Interestingly, 11 molecules showed binding free energy better than co-crystallized ligand (<−5.5254 kcal/mol for co-crystallized ligand), even though only 3 molecules (Amphibine B., Sativanine A., and Zizyphine F.) showed bonding interactions with amino acid residues of 2AZ5 active site, as shown in Figure 11 and Table 3).

On the other hand, only 4 molecules showed better interaction energy than co-crystallized ligand within transforming growth factor beta receptor type 1 kinase domain (TGFBR1 PDB ID: 6B8Y, resolution 1.65 Å) active site, while the remaining molecules showed either comparable or lower binding free energy compared to co-crystallized ligand (Table 4).

**Figure 12:**
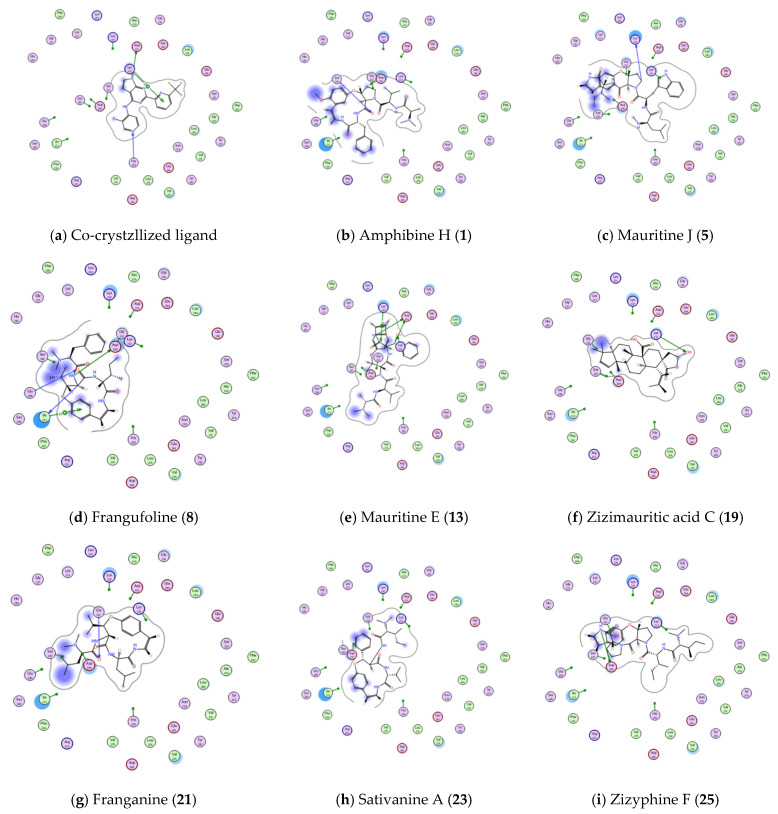
Showing 2D-binding interactions of co-crystallized ligand (**a**) and molecules **1**, **5**, **8**, **13**, **19**, **21**, **23**, **25** (**b**–**i**, respectively) within active sites of TGFBR1 kinase (PDB ID: 6B8Y).

Even though, 8 molecules succeed to show bonding interactions with amino acid residues of 6B8Y actives site as shown in Figure 12 and Table 4.

**Figure 13:**
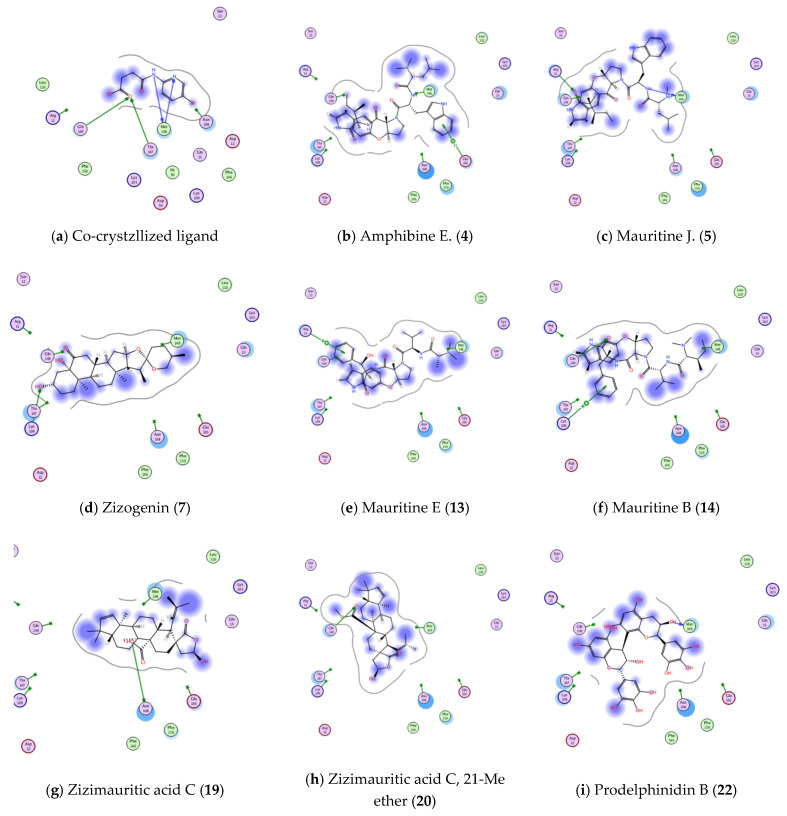
Showing 2D-binding interactions of co-crystallized ligand (**a**) and molecules **4**, **5**, **7**, **13**, **14**, **19**, **20**, **22** ((**b**–**i**), respectively) within active sites of Interleukin 1β (PDB ID: 6Y8M).

Finally, docking poses within Interleukin 1 beta (1L b PDB ID: 6Y8M, resolution 1.9 Å) active site showed better and promising results more than previous active sites; 12 molecules showed better binding scores than co-crystallized ligand (>−4.2536 kcal/mol, Table 5). Out of these 12 molecules, 8 molecules succeed to show strong bonding interaction within 6Y8M active site (ash shown in Figure 13 and Table 5).

In short, the binding modes and free energies obtained for the isolated compounds during the molecular docking studies within active sites of TGFBR1, TNF-α, and IL-1β confirm in-vivo animal studies results, which manifested by the significant change in the mRNA expression of *TGF-β* (increased) and the inflammatory markers, TNF-α and IL-1β) (decreased), as shown in Table 6.

#### 3.4.2. Antioxidant Activity Prediction

**Figure 14:**
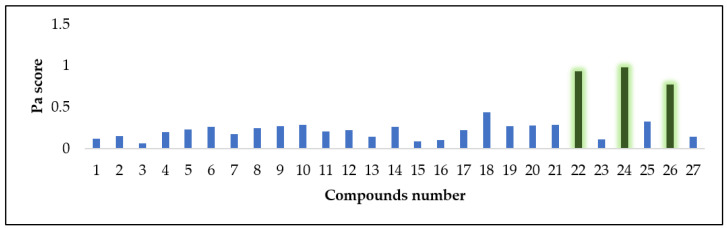
Predicted antioxidant activity scores for compounds **1**–**27** (Pa scores): Pa scores > 0.5 indicate high probability to show antioxidant activity in-vitro, while Pa scores < 0.5 indicate high probability to be an antioxidant agent in-vitro.

To predict the highly probable compounds with antioxidant or radical scavenging potential inside ZFE, we subjected the structures of all dereplicated compounds (**1**–**27**) to a neural networking based-biological activity prediction platform, namely PASS (Prediction of Activity Spectra of Substances) [67]. This platform applies a comprehensive pharmacophore-based virtual screening using a huge number of structures dataset with more than 3000 different biological activities. The retrieved result for each structure was provided as a score known as Pa score, as shown in Figure 14. Structures having a Pa score > 0.5 means having high probability to show antioxidant activity in-vitro, while those ones with a Pa scores < 0.5 means having no probability to be antioxidant agent. Accordingly, compounds 22, 24, and 26 are probably the ones responsible for the observed antioxidant activity of ZFE extract (Pa scores = 0.935, 0.979, and 0.775, respectively).

## 4. Conclusions

To the best of our knowledge, this is the first study to evaluate the wound healing potential ZFE along with the investigation of the chemical composition of this extract. In conclusion, ZFE displayed remarkable wound healing activity via accelerated wound closure rate, enhancing *TGF-β1*, VEGF, Type I collagen expression, and suppressing inflammatory markers (*TNF-α* and *IL-1β*). Furthermore, molecular docking of isolated compounds gave a putative prediction about the possible mode of the wound healing potential of ZFE extract through their efficient binding interactions within three wound healing-related proteins. Moreover, prediction of antioxidant activity of compounds with potential antioxidant activity was performed using PASS virtual screening platform. Finally, this study suggested the usage of ZFE in wound care as a promising therapy to accelerate wound healing. However, future detailed mechanistic studies are still required to confirm these predicted modes of action.

## Figures and Tables

**Table 1:** H_2_O_2_ radical scavenging activity of *Z. mauritiana* fruit extract.

Sample	IC_50_
*Zizyphus mauritiana* extract	189.2 ± 1.6 µg/mL
Ascorbic acid	194.2 ± 0.8 µg/mL

**Table 2:** Superoxide radical scavenging activity of *Z. mauritiana* fruit extracts.

Sample	IC_50_
*Zizyphus mauritiana* extract	146.7 ± 2.1 µg/mL
Ascorbic acid	154.4 ± 1.5 µg/mL

**Table 3:** Interaction binding energy (kcal/mol) and binding accuracy (RMSD; Å) of ZFE-derived compounds and co-crystallized ligand within TNFα active site (PDB ID: 2AZ5; 2.10 Å).

#	Molecule	Energy Score (kcal/mol)	RMSD (Å)
-	2AZ5 Co-crystallized ligand	−5.5254	1.3787
1	Amphibine H.	−6.6756	1.4343
2	Amphibine B.	−6.7857	2.0384
3	Amphibine D.	-	-
4	Amphibine E.	−5.9343	1.5565
5	Amphibine E., N-DeMe or Mauritine J.	−6.8964	1.6562
6	Amphibine F	-	-
7	Zizogenin or 2,6-Dihydroxyspirostane-3,12-dione	−5.7141	1.8788
8	Frangufoline	−6.1900	1.8342
11	Mauritine A.	-	-
12	Mauritine F.	-	-
13	Mauritine E.	−6.6522	1.7973
14	Mauritine B.	−6.4077	1.4182
15	Mauritine C.	-	-
16	Mauritine D.	-	-
17	Mauritine H.	−6.3098	1.6508
19	Zizimauritic acid C.	−5.5227	1.7836
20	Zizimauritic acid C., 21-Me ether	−5.2907	1.4763
21	Franganine	−5.7895	1.6281
22	Prodelphinidin B. or Gallocatechin(4α→8)gallocatechin	−4.9952	1.6455
23	Sativanine A.	−5.9033	1.9644
25	Zizyphine F.	−5.5143	2.0778

**Table 4:** Interaction binding energy (kcal/mol) and binding accuracy (RMSD; Å) of ZFE-derived compounds and the co-crystallized ligand within TGFBR1 kinase (PDB ID: 6B8Y; 1.65 Å).

#	Molecule	Energy Score (S; kcal/mol)	RMSD (Å)
-	6B8Y co-crystallized ligand	−5.102	1.1231
1	Amphibine H.	−6.6039	1.5284
2	Amphibine B.	-	-
3	Amphibine D.	-	-
4	Amphibine E.	-	-
5	Amphibine E., N-DeMe or Mauritine J.	−4.2228	1.5592
6	Amphibine F.	-	-
7	Zizogenin or 2,6-Dihydroxyspirostane-3,12-dione	−1.4649	1.5668
8	Frangufoline	−4.3323	1.5238
11	Mauritine A.	-	-
12	Mauritine F.	-	-
13	Mauritine E.	−1.8020	1.4485
14	Mauritine B.	-	-
15	Mauritine C.	-	-
16	Mauritine D.	-	-
17	Mauritine H.	−8.0128	1.3190
19	Zizimauritic acid C.	−3.9723	1.6145
20	Zizimauritic acid C., 21-Me ether	-	-
21	Franganine	−5.6339	1.3131
22	Prodelphinidin B. or Gallocatechin(4α→8)gallocatechin	−5.4270	1.2301
23	Sativanine A.	−3.9078	1.3504
25	Zizyphine F.	−4.9981	1.4643

**Table 5:** Interaction binding energy (S; kcal/mol) and binding accuracy (RMSD; Å) of ZFE-derived compounds and co-crystallized ligand within Interleukin 1β active site (PDB ID: 6Y8M; 1.90 Å).

#	Molecule	Energy Score (S; kcal/mol)	RMSD (Å)
-	6Y8M co-crystallized ligand	−4.2536	1.0950
1	Amphibine H.	−5.2842	1.5363
2	Amphibine B.	−4.8328	1.8557
3	Amphibine D.	-	-
4	Amphibine E.	−4.9107	1.8266
5	Amphibine E., N-DeMe or Mauritine J.	−5.4092	1.5702
6	Amphibine F.	-	-
7	Zizogenin or 2,6-Dihydroxyspirostane-3,12-dione	−4.3238	1.7200
8	Frangufoline	−5.3185	1.3011
11	Mauritine A.	-	-
12	Mauritine F.	-	-
13	Mauritine E.	−4.2738	1.8927
14	Mauritine B.	−5.6825	1.4982
15	Mauritine C.	-	-
16	Mauritine D.	-	-
17	Mauritine H.	-	-
19	Zizimauritic acid C.	−4.3469	1.4960
20	Zizimauritic acid C., 21-Me ether	−4.3448	1.7335
21	Franganine	−4.6395	1.3794
22	Prodelphinidin B. or Gallocatechin(4α→8)gallocatechin	−3.8771	1.2185
23	Sativanine A.	-	-
25	Zizyphine F.	−4.6249	1.9028

**Table 6:** Binding free energy score (S; kcal/mol) and binding interactions for different co-crystallized ligands and ZFE-derived compounds within TNFα (PDB ID: 2AZ5); TGFBR1 kinase (PDB ID: 6B8Y); and Interleukin 1β (PDB ID: 6Y8M) active sites.

Active Site	Ligand	Binding Energy Score (S; kcal/mol)	Ligand—Active Site Interactions		
			a. a. Residue	Bond Type	Bond Length (Å)
TNFα (PDB ID: 2AZ5)	Co-crystallized ligand	−5.5254	GLN 61	H-donor	2.97
			TYR 119	H-pi	4.08
	Amphibine B.	−6.7857	TYR 59	pi-H	4.02
	Sativanine A.	−5.9033	TYR 119	H-pi	3.95
	Zizyphine F.	−5.5143	TYR 59	pi-pi	3.85
TGFBR1 kinase (PDB ID: 6B8Y)	Co-crystallized ligand	−5.102	ASP 351	H-donor	2.72
			HIS 283	H-acceptor	2.89
			LYS 232	pi-H	3.94
	Amphibine H.	−6.6039	SER 287	H-acceptor	3.07
	Mauritine J.	−5.4092	LYS 337	H-donor	3.12
	Frangufoline	−4.3323	ILE 211	H-donor	3.22
			ASP 290	H-donor	3.07
			GLY 286	H-acceptor	3.43
			ILE 211	pi-H	4.35
	Mauritine E.	−1.8020	ASP 351	H-donor	3.35
			ASP 351	H-donor	2.88
			LYS 232	H-acceptor	3.00
			LYS 337	pi-H	3.89
	Zizimauritic acid C.	−3.9723	ASP 290	H-donor	3.39
	Franganine	−5.6339	LYS 232	H-acceptor	2.70
	Sativanine A.	−3.9078	GLY 212	H-acceptor	3.22
	Zizyphine F.	−4.9981	ASP 290	H-donor	3.13
			ASP290	H-donor	2.92
Interleukin 1β (PDB ID: 6Y8M)	Co-crystallized ligand	−4.2536	MET 148	H-donor	2.73
			MET 148	H-acceptor	2.94
			THR 147	H-acceptor	2.62
			GLN 149	H-acceptor	2.46
	Amphibine E.	−4.9107	GLU 105	pi-H	4.25
	Mauritine J.	−5.4092	MET 148	H-donor	3.20
			ARG 11	H-acceptor	2.97
	Zizogenin	−4.3238	LYS 109	H-acceptor	3.09
	Mauritine E.	−4.2738	ARG 11	pi-cation	4.34
	Mauritine B.	−5.6825	GLN 149	H-acceptor	3.32
			LYS 109	pi-cation	4.06
	Zizimauritic acid C.	−4.3469	ASN 108	H-donor	2.81
	Zizimauritic acid C., 21-Me ether	−4.3448	GLN 149	H-acceptor	3.04
	Prodelphinidin B.	−4.8631	MET 148	H-donor	2.76

## Data Availability

Not applicable.

## Supplementary Materials

The following supporting information can be downloaded at: https://www.mdpi.com/article/10.3390/plants11111392/s1, Figure S1: Histograms of wounded skin 7 days after incision; Group I [untreated]“1A”: showing the wound with underlying sloughed granulation tissue with compact and irregular collagen bundles(star). Group II [Z. mauritiana fruits extract]“1B”: showing granulation tissue filing the base of the defect from below is mainly cellular and collagen bundles appear as disorganized coarse and wavy bundles (stars). Group III [MEBOÒ] “1C”: showing collagen bundles packing the defect in a reticular pattern resembling that of the adjacent normal dermis (star) using Masson trichrome stain × 400, Figure S2: Histogram of wounded skin 14 days after incision; Group I [untreated] “2A”: showing the wide wound area, heavy inflammatory cellular infiltration and compact irregular collagen bundles (star); Group II [Z. mauritiana fruits extract] “2B”: showing typical stratified squamous keratinized epithelium and dermal matrix with coarse wavy collagen bundles in different directions (stars), and the newly formed hair follicles; Group III [MEBOÒ] “2C”: showing typical epithelium, thin scar tissue extending into the dermis, reticular dermis has coarse wavy collagen bundles arranged in different directions using Masson trichrome stain × 400, Table S1: The primer sequences of studied genes, Table S2. Dereplicated identified compounds from *Zizyphus mauritiana* fruits extract [53,54,55,56,57,58,59,60,61,62,63,64,65,66,68,69,70,71].
