# Supplementary material for: Wound Healing and Antioxidant Capabilities of Zizyphus mauritiana Fruits: In-Vitro, In-Vivo, and Molecular Modeling Study
Source: Plants (Basel). 2022 May 24;11(11):1392. doi: 10.3390/plants11111392 (PMC9183023; doi:10.3390/plants11111392)
Supplement: Supplementary file 1 [file plants-11-01392-s001.zip › plants-1705899-supplementary.pdf]

<u>**SUPPLEMENTARY DATA**</u>

Wound Healing and Antioxidant Capabilities of *Zizyphus mauritiana* Fruits. *In-vitro*, *In-vivo*, and Molecular Modeling Study

Nourhan Hisham Shady[1], Raya Soltane[2,3], Sherif A. Maher [4] , Entesar Ali Saber [5], Mahmoud A. Elrehany [4, 6], Yaser A. Mostafa[7] , Ahmed M. Sayed[8] and Usama Ramadan Abdelmohsen [1,9]*

[1] Department of Pharmacognosy, Faculty of Pharmacy, Deraya University, Universities Zone, P.O. Box 61111 New Minia City, Minia, Egypt.
[2] Department of Basic Sciences, Adham University College, Umm Al-Qura University, Makkah 21955, Saudi Arabia ; Email : rasoltan@uqu.edu.sa.
[3] Department of Biology, Faculty of Sciences, Tunis El Manar University, Tunis 1068, Tunisia.
[4] Department of Biochemistry, Faculty of Pharmacy, Deraya University, Universities Zone, New Minia City 61111, Egypt.
[5] Department of Histology and Cell Biology, Faculty of Medicine, Minia University, Minia, 61519 Egypt, Delegated to Deraya University, Universities Zone, New Minia City 61111, Egypt.
[6] Department of Biochemistry, Faculty of Medicine, Minia University, Minia 61519, Egypt.
[7] Pharmaceutical Organic Chemistry Department, Faculty of Pharmacy, Assiut University, 71526 Assiut, Egypt.
[8] Department of Pharmacognosy, Faculty of Pharmacy, Nahda University, Beni-Suef 62513, Egypt
[9] Department of Pharmacognosy, Faculty of Pharmacy, Minia University, 61519 Minia, Egypt.
* Correspondence: usama.ramadan@ mu.edu.eg, Tel.: +2-86-2347759, Fax: +2-86-2369075 (URA)

2. Materials and Methods

2.1. Plant Material Preparation

One kilogram of dried *Zizyphus mauritiana* fruits were obtained. It was extracted by maceration in 70% methanol at room temperature three times until exhausted. The alcoholic extract was concentrated under vacuum to yield a viscous residue (**100 g**). 10 g of the crude extract was used for *in vivo* study of the wound models by dissolving *Zizyphus mauritiana* fruits extract in distilled water (2 gm of crude extract in 100 mL of distilled water) and stored at 4°C in the dark.

.2.2. *In-Vitro* Antioxidant Activity

2.2.1. Hydrogen Peroxide Scavenging Activity

The reaction with a defined amount of exogenously provided H_2O_2 was used to determine the hydrogen peroxide (H_2O_2) scavenging activity that reflects the anti-oxidative capacity of ZFE extract. Colorimetric analysis was used to estimate the residual H_2O_2 [1]. In brief, 20µl of the sample was mixed with 500 µl of H_2O_2 and incubated at 37ºC for 10 minutes. After that, 500 µl of enzyme/3, 5-dichloro-2-hydroxyl-benzenesulfonate solution was added and incubated at 37ºC for 5 minutes. Colorimetrically, the intensity of the colored product was measured at 510 nm. Ascorbic acid was used as a positive control. By comparing the percentages of H_2O_2 scavenging activity was determined by comparing the results of the ZFE test sample with those of the control using the following formula:

$$\text{scavenging activity} = \frac{\text{A control} - \text{A sample}}{\text{A control}} \times 100$$

IC_{50} of each sample was calculated after performing the assay at four different concentrations using Graph pad prism 7 software.

2.2.2. Superoxide Radical Scavenging Activity

The superoxide anion scavenging activity was measured as described by Srinivasan R. *et al.*, **2007** [2]. The superoxide anion radicals were formed in a Tris-HCl buffer (16 mM, pH 8.0) containing 90 µl of NBT (0.3 mM), 90 µl of NADH (0.936 mM), 0.1 ml of ZFE extract (125, 250, 500, and 1000 µg/mL), and 0.8 mL Tris- HCl buffer (16 mM, PH 8.0). The reaction was initiated by adding 0.1 ml PMS solution (0.12 mM) to the mixture, which was then incubated at 25˚C for 5 minutes, and measured at 560 nm, the absorbance was measured. Ascorbic acid was selected as a reference. The percentage inhibition was obtained by comparing the test findings to those of the control using the formula below:

$$\text{Superoxide scavenging activity} = \frac{\text{A control} - \text{A sample}}{\text{A control}} \times 100$$

IC_{50} was calculated using Graph pad prism 7 software by performing the test at four different concentrations.

2.3. *In-Vivo* Wound healing Activity

Study Animals

Twenty-four adult male New Zealand Dutch strain albino rabbits were purchased from the Faculty of pharmacy, Deraya University, Minia, Egypt having an age of 6 months weighing from 1 to 1.2 kg. The rabbits were kept in separate cages and were given a standard diet and tap water under controlled settings of 25 °C temperature and 55% humidity with twelve hours cycle of dark and light. Extreme care was taken and all procedures on rabbits were adopted according to Deraya laboratory guidelines. **The approval code is 8/2021.**

Wound Excision Model

The rabbits were anesthetized by intraperitoneal (I.P.) injection with ketamine (Alphasam company®, Holland, 50 mg/kg) and xylazine hydrochloride (Alphasam company®, Holland, 10 mg/kg [3]. After anesthesia, the awareness (alertness) level of rabbits was determined and shaving was done. The shaving area was back of the animal, in the withers. Anticipation was done by alcohol 70% and povidone-iodine 10% 7 times. The animals were depilated on the paravertebral area before wound creation and a circular excision wound of 6 mm in diameter was created using a biopsy punch [4]. This procedure generates the wound in both the epidermis and the dermis

layers. Using experimental rabbits 3 groups were formed, each group include 8 rabbits. The rabbits of **Group 1** did not receive any treatment (bare wound) and were used as the negative control. Wounds in **Group 2** were treated with *Zizyphus mauritiana* fruits extract (2 mg/wound), while **Group 3** was treated with MEBO® ointment (100 mg/wound) and were used as positive control (Market treatment). The wounded area was covered with a standard surgical dressing while redressing was performed with fresh dressing on 3, 7 and 10 days.

2.3.1. Wound Closure Rate Estimation

For the physical appearance and closure of wounds, photographs of wounded areas were taken by using a digital camera (DSC-W320 Sony; Sony Corp., Tokyo, Japan) on 0, 3, 7, 10, and 14 days posing vertically to middle of wound with a distance of 6 cm. The reduction in wounded area (wound contraction) was used as an indicator of efficacy of the treatment. Thus, the periphery of the excisional wound was outlined after creating the wound with the help of transparent paper. The contraction in wounds was recorded on 3, 7, 10, and 14 days and expressed as a percent of the healed wounded area. The percentage wound contraction was estimated using the formula:

"Wound contraction = Area of original wound-Area at nth day/ Area of original wound x 100 %"

Finally, the day of complete wound healing (epithelialization) of each wound was observed for all 3 groups.

2.3.2. Effect on Expression of *TGF-β, TNF-α, IL-1β*, collagen I, and VEGF

2.3.2.1. Total RNA extraction

About 50 mg of dorsal skin tissues was homogenized by ultrasonic homogenizer (Sonics-Vibracell, Sonics& Materials Inc., Newtown, USA) in 0.5 ml TRIzol TM reagent (Amresco, Solon, USA). Total RNA was extracted from dorsal skin tissues using TRIzol RNA extraction reagent (Amresco, Solon, USA) following the manufacturer's instructions. The total RNA concentration was determined at a 260 nm and the purity was calculated according to the ratio A260/A280. Samples have a purity ≥ 1.7 were used for qRT-PCR using GAPDH (Glyceraldehyde-3-phosphate dehydrogenase) as a reference housekeeping gene.

2.3.2.2. Real-time qRT-PCR

cDNA synthesis was performed for equal quantities of total RNA in all samples using the RevertAid H Minus First Strand cDNA Synthesis kit (#K1632, Thermo Scientific Fermentas, St. Leon-Ro, Germany) according to the manufacturer's instructions. Real-time PCR was carried out with single-stranded cDNAs. PCR reactions were performed by SYBER Green [#K0251, Thermo Scientific Fermentas St. Leon-Ro, Germany-Maxima SYBER Green qPCR Master Mix (2X)] using StepOne Real-Time PCR Detection System (Applied Biosystems). The set of primers used for Real-Time PCR were mentioned in **Table 2**. Real-time polymerase chain reaction (qRT-PCR) was carried out using 20 µl of RealMOD Green qRT-PCR Mix kit (iNtRON biotechnology) with 0.02 µg RNA

per reaction containing 10 Pmol of specific primers, for 30 cycles of 95°C for 10 sec. and 60°C for 1 min. Comparative Ct (threshold cycle) method was used to determine the relative amounts of the products. The relative expression was calculated using the formula $2^{(-\Delta\Delta Ct)}$ [5]. They were scaled relative to controls where control samples were set at a value of 1.

Table S1. The primer sequences of studied genes

Gene name	GenBank accession		
IL- β1	NC_013670.1	Forward	5'-AGCTTCTCCAGAGCCACAAC-3'
		Reverse	5'-CCTGACTACCCTCACGCACC-3'
GAPDH	NC_013676.1	Forward	5'-GTCAAGGCTGAGAACGGGAA-3'
		Reverse	5'-ACAAGAGAGTTGGCTGGGTG-3'
TGF-β1	NC_013672.1	Forward	5'-GACTGTGCGTTTTGGGTTCC-3'
		Reverse	5'-CCTGGGCTCCTCCTAGAGTT-3'
TNF-α	NC_013680.1	Forward	5'-GAGAACCCCACGGCTAGATG-3'
		Reverse	5'-TTCTCCAACTGGAAGACGCC-3'

2.3.2.3. Western Blotting

For direct immunoblotting, tissue sections were homogenized in lysis buffer (20 mM Tris, 1 mM EDTA, 100 mM NaCl, protease inhibitors mixture, and 0.5% Triton X-100 buffer) then protein concentrations were determined by Bradford method. Tissue homogenates (50 µg of total proteins) were loaded on 12% sodium dodecyl sulfate-polyacrylamide gel electrophoresis (SDS-PAGE) running for 1 h., at 100 voltage. Following electrophoresis, proteins were transferred to polyvinylidene fluoride (PVDF) membranes. After blocking for 1h., in a Tris-buffered saline (TBS-T) blocking solution containing 5% (w/v) non-fat milk and 0.05% tween-20, they were incubated with primary antibodies for collagen I, VEGF and *β-actin* (1:1000; Santa Cruz Biotechnology, Santa Cruz, CA) overnight at 4°C. Horseradish peroxidase-conjugated polyclonal goat anti-rabbit immunoglobulin (Cell Signaling Technology Inc., MA, USA) was used as a secondary antibody at a dilution of 1:5000 in blocking buffer. Bands were visualized by chemiluminescence, using an enhanced chemiluminescence kit (ECL, GE Healthcare, Chicago, IL, USA), according to the manufacturer's instructions and detected using an analyzer for luminescent images (LAS-4000, Fujifilm Co., Tokyo, Japan). Bands corresponding to proteins of the different groups were accessed relative to the normal control group densitometrical after normalization to *β*-actin using Image J Software.

2.4. Histopathological analysis

Rabbits were anesthetized on 7th and 14th days and the wounded area with a periphery of about 5 mm of ambient un-wounded skin biopsy was taken. These skin tissues were fixed in a 10 % formalin solution for 2-3 days followed by tissue processing and embedding in paraffin. Thin tissue sections of 5 µm thickness were made using Microtome and stained with hematoxylin and eosin stain (H&E) and Masson trichrome staining. The stained sections were observed using a light microscope fitted with a camera about neovascularization, epidermis, scar, and granulation tissues, and images were taken.

Data Statistical Analysis

The statistical analysis was performed using GraphPad Prism (LaJolla, CA). The Shapiro Wilk test for normality of variance and then nonlinear fit of normalized variables were performed. Leven's test for homogeneity of variance was performed and finally Two-way ANOVA was performed. The results were represented as mean ± standard deviation (SD). Two-way ANOVA was applied to determine whether the results have significant variations and a P-value ≤ 0.05 was considered significant.

2.5. LC-MS Metabolomics Analysis

Metabolomic profiling was performed on the crude extract of *Zizyphus mauritiana* fruits on an Acquity Ultra Performance Liquid Chromatography system coupled to a Synapt G2 HDMS quadrupole time-of-flight hybrid mass spectrometer (Waters, Milford, CT, USA). Chromatographic separation was carried out on a BEH C18 column (2.1 × 100 mm, 1.7 µm particle size; Waters, Milford, CT, USA) with a guard column (2.1 × 5 mm, 1.7 µm particle size) and a linear binary solvent gradient of 0–100% eluent B over 6 min at a flow rate of 0.3 mL. min^{-1}, using 0.1% formic acid in water (v/v) as solvent A and acetonitrile as solvent B. The injection volume was 2 µL and the column temperature was 40 °C. MS converter software was used in order to convert the raw data into divided positive and negative ionization files. Obtained files were then subjected to the data mining software MZmine 2.10 (Okinawa Institute of Science and Technology Graduate University, Japan) for deconvolution, peak picking, alignment, deisotoping, and formula prediction. The databases used for the identification of compounds were: MarinLit: http://pubs.rsc.org/marinlit/, and Dictionary of Natural Products (DNP) 2018: http://dnp.chemnetbase.com/faces/chemical/ChemicalSearch.xhtml

Results and discussion

Table S2. Dereplicated identified compounds from *Zizyphus mauritiana* fruits extract.

No	Name	Structure	Retention Time	Source	Exact mass	Ref

1	Amphibine H	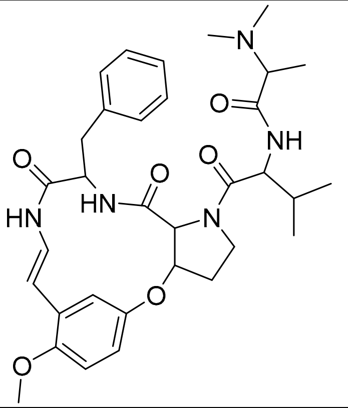	13.19	*Zizyphus spina-christi*	605.321	[3]
2	Amphibine B	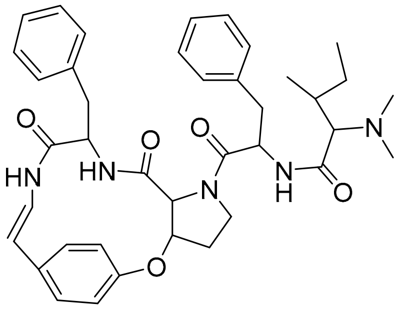	13.21	*Zizyphus mauritiana*	655.3577	[4]
3	Amphibine D	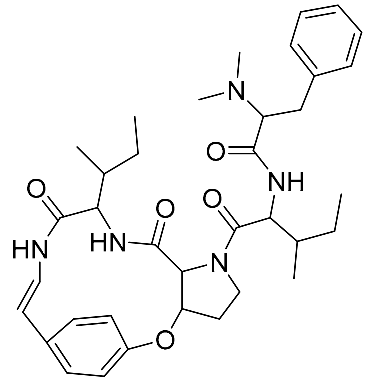	16.02	*Zizyphus mauritiana*	631.3733	[4]
4	Amphibine E	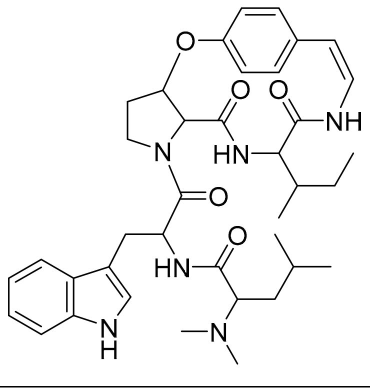	13.87	*Zizyphus mauritiana*	670.3842	[4]

5	Mauritine J.	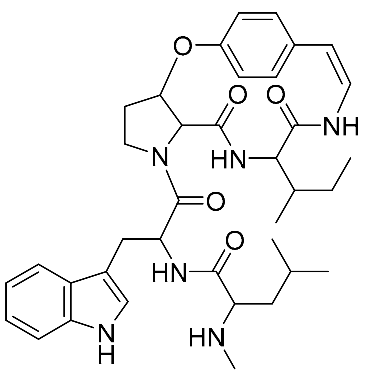	15.93	*Zizyphus mauritiana*	656.3686	[5]
6	Amphibine F	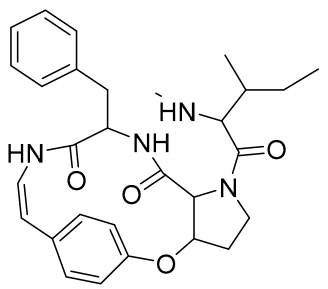	8.31	*Zizyphus mauritiana*	504.2736	[6]
7	Zizogenin	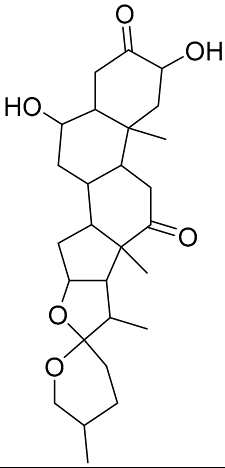	11.62	*Zizyphus mauritiana*	460.2824	[7]
8	Frangufoline	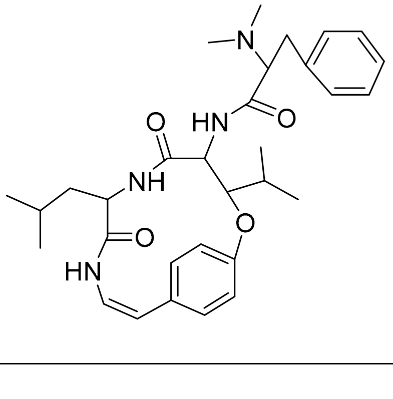	3.75	*Zizyphus mauritiana*	534.3206	[6]

9	12-Hentriacontanol (ξ)-form	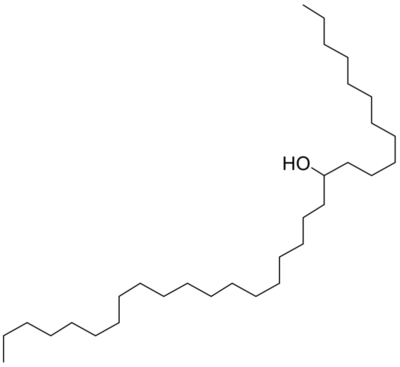	16.38	*Zizyphus mauritiana*	452.4957	[8]
10	Hexadecanoic acid Undecyl ester	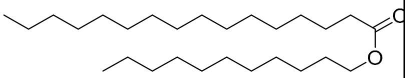	7.35	*Zizyphus mauritiana*	410.4123	[9]
11	12-Hydroxy-9-tetratriacontanone	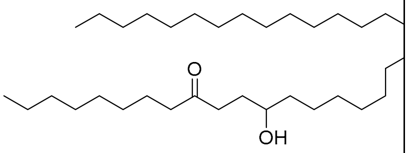	12.83	*Zizyphus mauritiana*	508.5219	[8]<br>[8]
12	Mauritine A	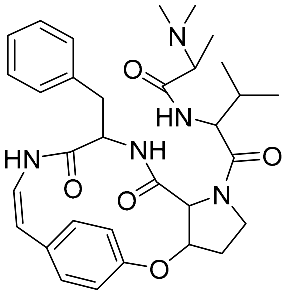	13.987	*Zizyphus mauritiana*	575.3107	[7]

13	Mauritine F.	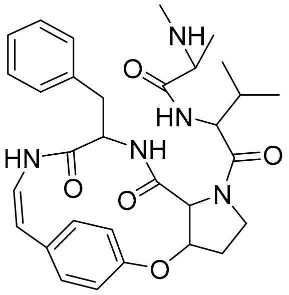	9.69	*Zizyphus mauritiana*	561.301	[10]
14	Mauritine E.	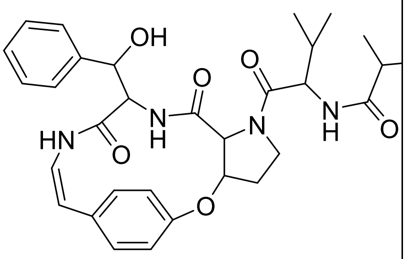	9.56	*Zizyphus mauritiana*	591.305	[11]
15	Mauritine B	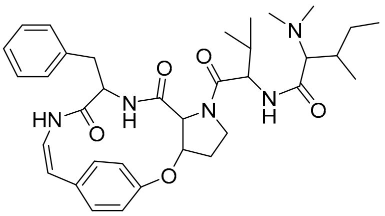	8.93	*Zizyphus mauritiana*	617.357	[6]
16	Mauritine C	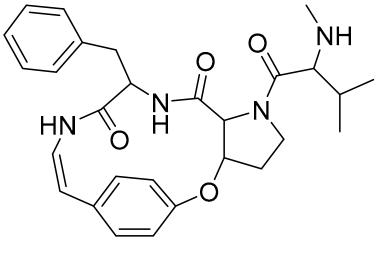	9.12	*Zizyphus mauritiana*	490.258	[6]

17	Mauritine D	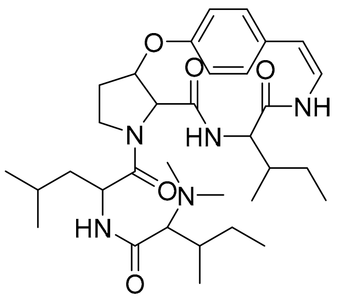	16.47	*Zizyphus mauritiana*	597.38902	[6]
18	Mauritine H	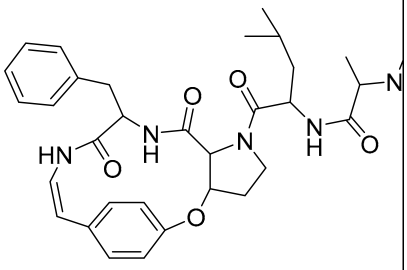	12.62	*Zizyphus mauritiana*	589.3264	[10]
19	3,6,8-Trihydroxy-1-methylanthraquinone-2-carboxylic acid	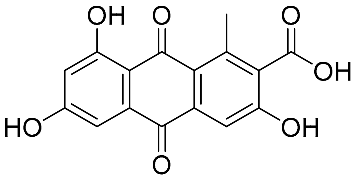	6.28	*Zizyphus mauritiana*	314.0426	[12]
20	Zizimauritic acid C	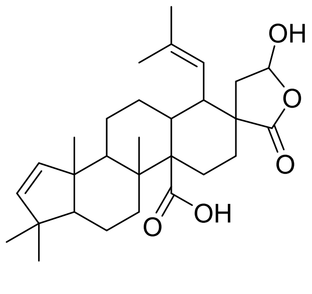	3.38	*Zizyphus mauritiana*	470.3032	[13]

21	Zizimauritic acid C 21-Me ether	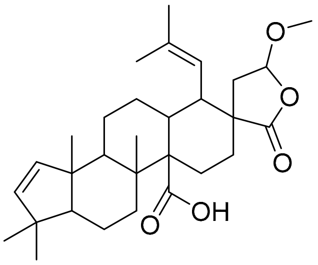	13.71	*Zizyphus mauritiana*	484.3188	[13]
22	Franganine	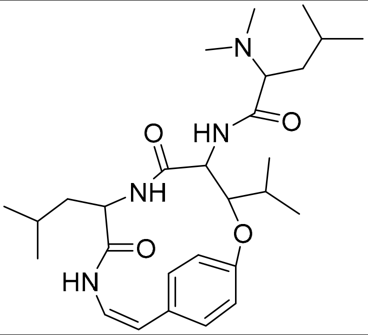	8.16	*Zizyphus mauritiana*	500.336	[14]
**23**	Gallocatechin-(4α→8)-gallocatechin. Or Prodelphinidin B	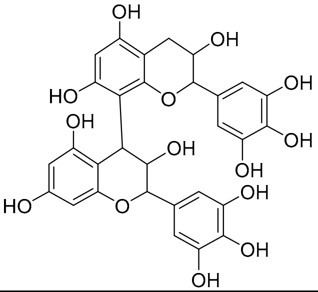	3.27	*Zizyphus spina-christi*	610.310	[15]
24	Sativanine A	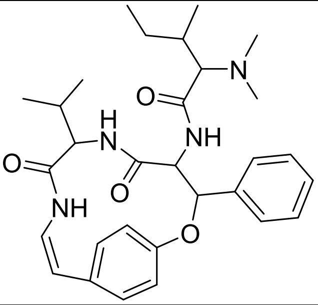	8.82	*Zizyphus spina-christi*	520.3049	[10]
**25**	Zizyflavoside B.	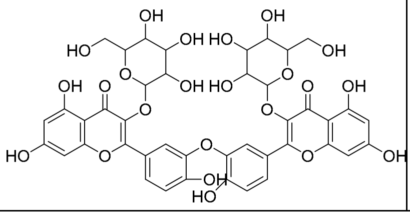	9.83	*Zizyphus spina-christi*	910.1803	[16]

**26**	Zizyphine F	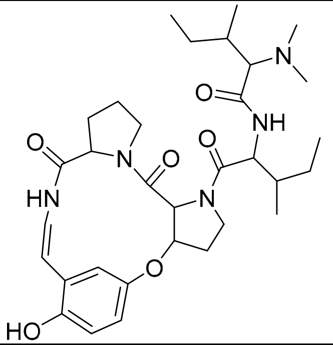	16.55	*Zizyphus spina-christi*	597.3526	[3]
**27**	3',4',5,7-Tetrahydroxyflavone	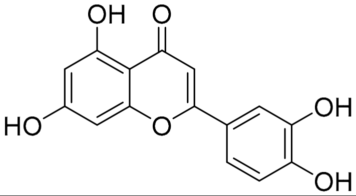	3.37	*Zizyphus oenoplia*	286.239	[17]
**28**	Jujubasaponin IV	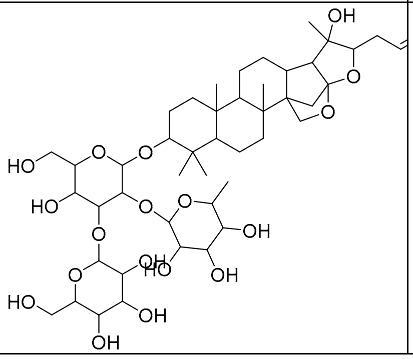	9.83	*Zizyphus jujuba*	942.510	[18]<br>[18]

3.2.3. Histopathological Study

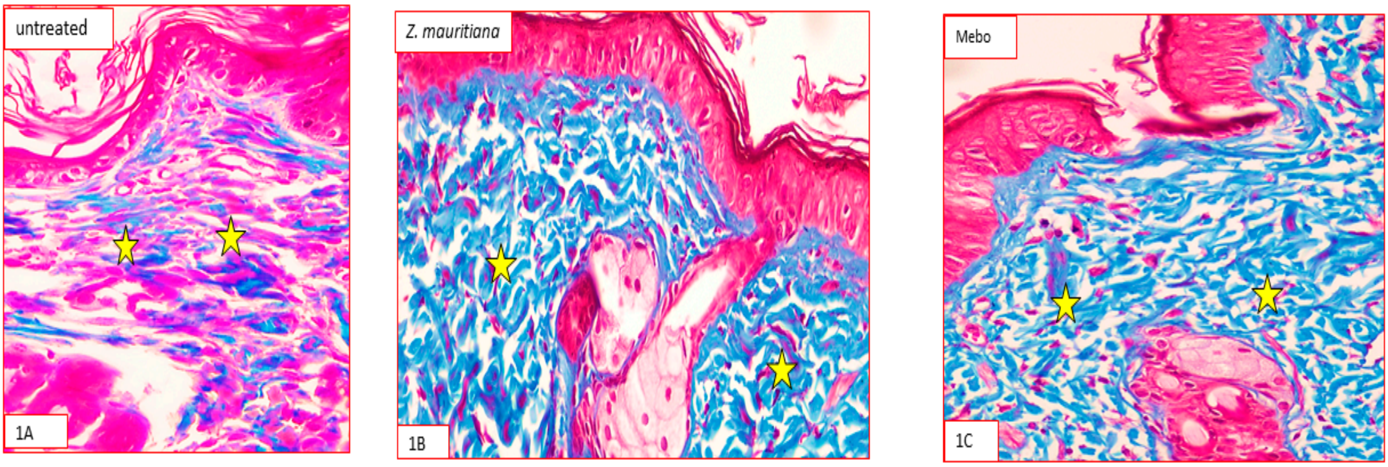

Fig. S1. Histograms of wounded skin 7 days after incision; Group I [untreated]"**1A**": showing the wound with underlying sloughed granulation tissue with compact and irregular collagen bundles(star). Group II [*Z. mauritiana* fruits extract]"**1B**": showing granulation tissue filing the base of the defect from below is mainly cellular and collagen bundles appear as disorganized coarse and wavy bundles (stars). Group III [MEBO®] "**1C**": showing collagen bundles packing the defect in a reticular pattern resembling that of the adjacent normal dermis (star) using **Masson trichrome stain x 400.**

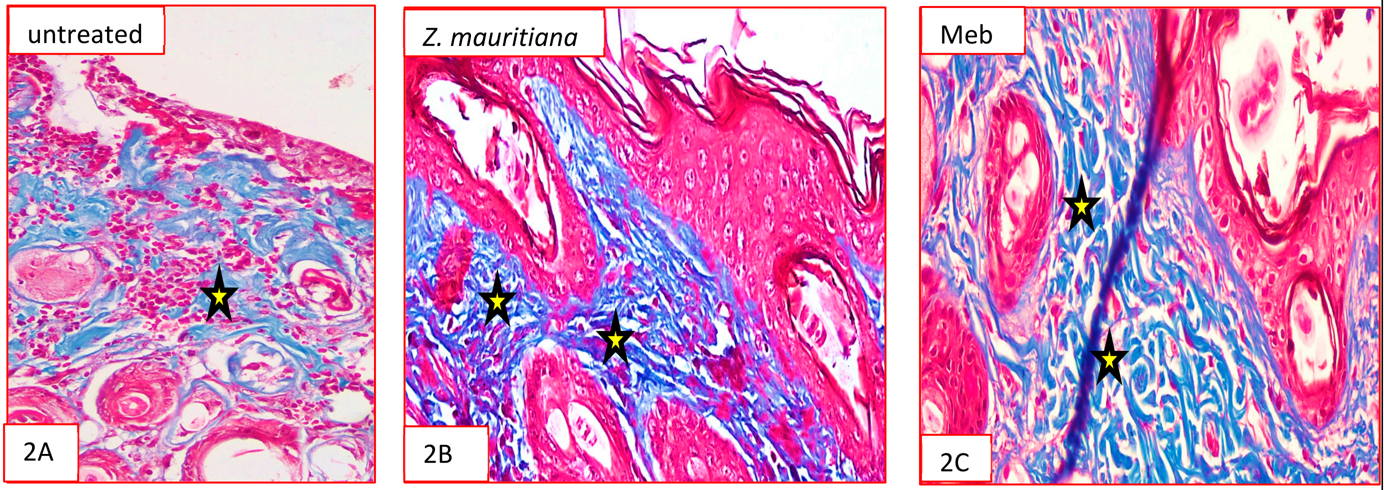

Fig. S2. Histogram of wounded skin 14 days after incision; Group I [untreated] "2A": showing the wide wound area, heavy inflammatory cellular infiltration and compact irregular collagen bundles (star); Group II [*Z. mauritiana fruits extract*] "2B": showing typical stratified squamous keratinized epithelium and dermal matrix with coarse wavy collagen bundles in different directions (stars), and the newly formed hair follicles; Group III [MEBO®] "2C": showing typical epithelium, thin scar tissue extending into the dermis, reticular dermis has coarse wavy collagen bundles arranged in different directions using **Masson trichrome stain x 400.**

References

1. Hassan, H.; Abdel-Aziz, A. Evaluation of free radical-scavenging and anti-oxidant properties of black berry against fluoride toxicity in rats. *Food Chem. Toxicol.* **2010**, *48*, 1999–2004, https://doi.org/10.1016/j.fct.2010.05.018.
2. Srinivasan, R.; Chandrasekar, M.; Nanjan, M.J.; Suresh, B. Antioxidant activity of Caesalpinia digyna root. *J. Ethnopharmacol.* **2007**, *113*, 284–291, https://doi.org/10.1016/j.jep.2007.06.006.
3. Abdel-Galil, F.M.; El-Jissry, M.A. Cyclopeptide alkaloids from Zizyphus spina-christi. *Phytochemistry* **1991**, *30*, 1348–1349, https://doi.org/10.1016/s0031-9422(00)95238-5.
4. Tschesche, R.; Kaußmann, E.U.; Fehlhaber, H. Alkaloide aus Rhamnaceen, XIII. Amphibin-B, -C, -D und -E, vier Peptidalkaloide aus Ziziphus amphibia A. Cheval. *Eur. J. Inorg. Chem.* **1972**, *105*, 3094–3105, https://doi.org/10.1002/cber.19721050932.
5. Jossang, A.; Zahir, A.; Diakite, D. Mauritine J, a cyclopeptide alkaloid from Zizyphus mauritiana. *Phytochemistry* **1996**, *42*, 565–567, https://doi.org/10.1016/0031-9422(95)00965-5.
6. Panseeta, P.; Lomchoey, K.; Prabpai, S.; Kongsaeree, P.; Suksamrarn, A.; Ruchirawat, S.; Suksamrarn, S. Antiplasmodial and antimycobacterial cyclopeptide alkaloids from the root of Ziziphus mauritiana. *Phytochemistry* **2011**, *72*, 909–915, https://doi.org/10.1016/j.phytochem.2011.03.003.
7. Tripathi, S. Ziziphus jujuba: A phytopharmacological review. *Int. J. Res. Dev. Pharm. Life Sci.* **2014**, *3*, 959–966.
8. Alves, R.J.V.; Pinto, A.C.; Da Costa, A.V.M.; Rezende, C.M. Zizyphus mauritiana Lam. (Rhamnaceae) and the chemical composition of its floral fecal odor. *J. Braz. Chem. Soc.* **2005**, *16*, 654–656, https://doi.org/10.1590/s0103-50532005000400027.
9. Agarwal, S.K.; Verma, S.; Singh, S.S.; Sammal, S.S.; Kumar, S. ChemInform Abstract: New Aliphatic Ester and Alcohol from the Leaves of Ziziphus mauritiana.. *ChemInform* **2010**, *33*, https://doi.org/10.1002/chin.200232224.
10. Goyal, M.; Sasmal, D.; Nagori, B. Review on Ethnomedicinal uses, Pharmacological activity and Phytochemical constituents of Ziziphus mauritiana (Z. jujuba Lam., non Mill). *Spatula DD - Peer Rev. J. Complement. Med. Drug Discov.* **2012**, *2*, 107–116, https://doi.org/10.5455/spatula.20120422080614.
11. Tschesche, R.; Wilhelm, H.; Kaußmann, E.U.; Eckhardt, G. Alkaloide aus Rhamnaceen, XVII1) Mauritin-C,-D,-E und-F; neue Peptidalkaloide aus Ziziphus mauritiana Lam. *Justus Liebigs Ann.* Der *Chem.* **1974**, *1974*, 1694-1701.
12. Afzal, S.; Batool, M.; Ch, B.A.; Ahmad, A.; Uzair, M.; Afzal, K. Immunomodulatory, Cytotoxicity, and Antioxidant Activities of Roots of Ziziphus mauritiana. *Pharmacogn. Mag.* **2017**, *13*, S262–S265, https://doi.org/10.4103/pm.pm_398_16.
13. Ji, C.-J.; Zeng, G.-Z.; Han, J.; He, W.-J.; Zhang, Y.-M.; Tan, N.-H. Zizimauritic acids A–C, three novel nortriterpenes from Ziziphus mauritiana. *Bioorganic Med. Chem. Lett.* **2012**, *22*, 6377–6380, https://doi.org/10.1016/j.bmcl.2012.08.074.
14. Mahajan, R.; Chopda, M. Phyto-Pharmacology of Ziziphus jujuba Mill-A plant review. *Pharmacogn. Rev.* **2009**, *3*, 320.
15. Zozio, S.; Servent, A.; Cazal, G.; Mbéguié-A-Mbéguié, D.; Ravion, S.; Pallet, D.; Abel, H. Changes in antioxidant activity during the ripening of jujube (Ziziphus mauritiana Lamk). *Food Chem.* **2014**, *150*, 448–456, https://doi.org/10.1016/j.foodchem.2013.11.022.
16. Mostafa, M.; Takano, F.; Ohta, T. P-54 New biflavonoyl ether glycosides from Zizyphus spina-christi: Symmetrical ether derived from two different flavonoids. In *Symposium on the Chemistry of Natural Products, Symposium Papers 52*; Symposium on the Chemistry of Natural Products Steering Committee: Hokkaido, Japan, 2010.
17. Prashith, K.T.; Vinayaka, K.S.; Mallikarjun, N.; Bharath, A.C.; Shailendra, K.B.; Mc Rakesh, K.; Vinod, K.H. Antibacterial, Insecticidal and Free radical scavenging activity of methanol extract of Ziziphus rugosa Lam. (Rhamnaceae) fruit pericarp. *Pharmacogn. J.* **2011**, *2*, 65–69, https://doi.org/10.1016/s0975-3575(11)80028-3.
18. Yoshikawa, K.; Shimono, N.; Arihara, S. Antisweet Natural Products. VI. Jujubasaponins IV, V and Vi from Zizyphus jujuba MILL. *Chem. Pharm. Bull.* **1992**, *40*, 2275–2278.
